# Robust bilinear rotations II

**DOI:** 10.5194/mr-7-1-2026

**Published:** 2026-02-05

**Authors:** Yannik T. Woordes, Burkhard Luy

**Affiliations:** 1 Institute of Organic Chemistry and Institute for Biological Interfaces 4 – Magnetic Resonance, Karlsruhe Institute of Technology (KIT), Kaiserstr. 12, 76131 Karlsruhe, Germany

## Abstract

Bilinear rotations are essential building blocks in modern NMR spectroscopy. They allow the rotation of an isolated spin without couplings (i.e., bilinear interactions) in one way, while rotating spins with a matched coupling in another way. Different classes of rotations form the different bilinear rotations, with the acronyms BIRD, TANGO, BANGO, and BIG-BIRD. All original elements have in common hard pulses limiting bandwidths and defined rotations for coupled spins that are possible only for a narrow range of coupling constants. We recently introduced the COB-BIRD with a general optimization procedure to obtain robust bilinear rotations that are well compensated for couplings, offsets, and B_1_ inhomogeneities [Bibr bib1.bibx65]. Here we show a fundamental principle on how the COB-BIRD can be used to construct all types of bilinear rotations, with the same improved robustness covering a coupling range of 120–250 Hz. In addition, a construction principle for universal rotation pulses is adapted to produce bilinear rotations from INEPT-type transfer elements, allowing the construction of bilinear rotations also for higher coupling ranges from, for example, COB3-INEPT, with coupling compensation in the range of 120–750 Hz. After introducing the two fundamental design principles, example sequences of the four classes of bilinear rotations and different degrees of robustness are derived and characterized in theory and experiment. In addition, a highly useful HMBC/ASAP-HSQC-IPE-COSY supersequence is introduced with a (COB-)BANGO element for Ernst-angle-type excitation. Finally, BIRD-decoupled J -resolved INEPT experiments with extreme compensation for partially aligned samples, with total couplings ranging from 47 Hz up to 434 Hz, are demonstrated.

## 1 Introduction

Bilinear rotation elements represent fundamental building blocks in NMR spectroscopy, with a large variety of applications. The success story started with bilinear rotation decoupling (BIRD) by Garbow, Weitekamp, and Pines in 1982 [Bibr bib1.bibx18], with a spin system selective 180° rotation as the central element. A decade later, the basic element has been generalized with a systematic nomenclature by Uhrín, Liptaj, and Kövér [Bibr bib1.bibx62]. Since then, it has been used for spectral cleanup [Bibr bib1.bibx38], homonuclear decoupling [Bibr bib1.bibx37], enhanced measurement of couplings [Bibr bib1.bibx16], enhanced resolution in a J -evolved dimension [Bibr bib1.bibx17], and many more applications. Next to BIRD variants, several other bilinear rotations exist, such as the TANGO [Bibr bib1.bibx63] and BANGO [Bibr bib1.bibx54] universal coupling-dependent rotations and corresponding BIG-BIRD [Bibr bib1.bibx54] and TIG-BIRD [Bibr bib1.bibx7] elements that transfer initial polarization into any desired magnetization, depending on the absence or presence of a large one-bond heteronuclear coupling. The basic BIRD element has been extended for better robustness, leading to G-BIRD [Bibr bib1.bibx15] with enhanced cleanup, CAGE-BIRD [Bibr bib1.bibx36] for better homonuclear coupling handling, and BASEREX ([Bibr bib1.bibx24]; [Bibr bib1.bibx6]; [Bibr bib1.bibx53]) for the selective treatment of isotope-labeled samples. Nevertheless, all basic bilinear rotations have in common the fact that they are relatively sensitive to coupling mismatch and offset effects [Bibr bib1.bibx18]. The issue has been recognized early on, and J - and offset-compensated BIRD elements have already been proposed in the seminal BIRD paper [Bibr bib1.bibx18]. However, only a recent systematic study of correspondingly coupling-, offset-, and B_1_-compensated BIRD elements (COB-BIRD) has provided a robust element for isotropic and very weakly coupled partially aligned samples [Bibr bib1.bibx65].

In light of novel applications like fast-pulsing and interleaved acquisition sequences [Bibr bib1.bibx66], robust sequences are also needed for bilinear rotation elements beyond BIRD. In this article, we therefore derive a general scheme that reduces all bilinear rotations to its central spin system selective refocusing element, with which the robustness of the COB-BIRD can be transferred to all other bilinear rotations, i.e., robust COB-TANGO, COB-BANGO, COB-BIG-BIRD, and COB-BASEREX. In addition, we extend and apply a previously derived symmetry scheme [Bibr bib1.bibx41] to deduce sequences with J compensation over even larger ranges of couplings than COB-BIRD by using already existing compensated INEPT-type transfer elements [Bibr bib1.bibx11]. All derived elements are studied in both theory and experiment. We also present two applications demonstrating the usefulness of the extended J -coupling range and the benefit of a robust COB-BANGO element in modern NORD-type fast-pulsing supersequence schemes.

## 2 Generalizing COB-enhanced bilinear rotations

Bilinear rotations are spin system selective heteronuclear building blocks that distinguish spins I – which are not directly coupled to a heteronucleus – from spin systems IS , where the spin I is coupled to a spin S via a large heteronuclear coupling J . In BIRD elements, the difference between uncoupled and coupled spins usually lies in the phase of a transverse π rotation. TANGO elements, instead, provide a 90° (or any arbitrary β ) pulse for one and either 0° or 180° for the other type of spin system. BANGO elements allow universal rotations with arbitrary flip angles $\beta^{I}$ and $\beta^{IS}$ for the spin systems. While all these elements allow rotations only about a specific rotation axis, BIG-BIRD rotates initial $I_{z}$ polarization into any final position that can be reached by effective $\beta_{\varphi^{I}}^{I}$ and $\beta_{\varphi^{IS}}^{IS}$ rotations, introducing the effective phases $\varphi^{I}$ and $\varphi^{IS}$ for the two spin systems. As it turns out, on the one hand, the different types of bilinear rotations manipulate spins in very different ways, but, on the other hand, they all have an identical central building block in common that is responsible for their spin system selective properties (cf. Fig. [Fig F1]).

**Figure 1 F1:**
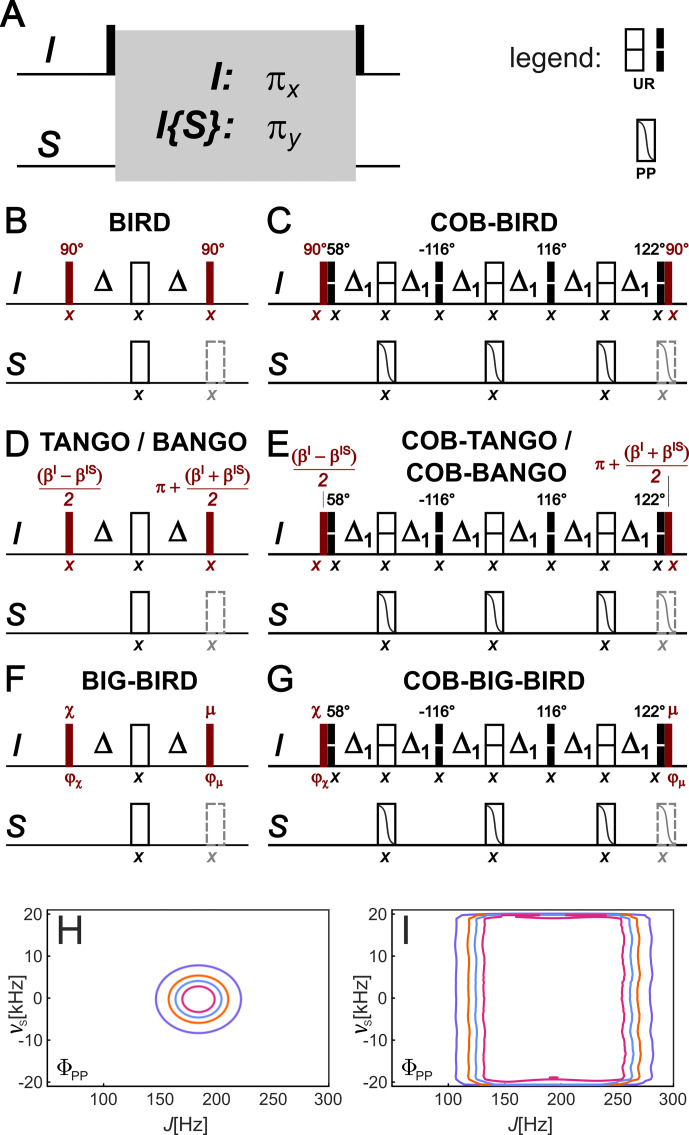
Construction of any type of bilinear rotation from a single spin system selective π rotation element. The underlying insight into the construction scheme is the fact that any of the basic bilinear rotations in literature is based on two flanking pulses (shown in dark red) around the very same rotation scheme that provides a $\pi_{x}$ rotation for an uncoupled spin I and a $\pi_{y}$ rotation for an IS spin system, with a coupling constant that is matched to sequence (**A**, rotation element represented by the gray box). Corresponding BIRD, TANGO, BANGO, and BIG-BIRD bilinear rotations are obtained by specifically chosen flanking pulses **(B, D, and F)**. If the simple refocused delay with Δ=1/(2J) is replaced by a more robust spin system selective π rotation element, like the one here derived from the recently published COB-BIRD bilinear rotation [Bibr bib1.bibx65], any type of bilinear rotation can be obtained by simply using corresponding flanking pulses with the element **(C, E, and G)**. Be aware that consecutive pulses before and after the rotation element – consisting of the red flanking pulses and the first/last pulse of the central element – are represented for clarity as separate pulses but may be combined to a single pulse (or universal rotation pulse shape) in a particular application. Also note the convention for point-to-point (PP)- and universal rotation (UR)-shaped pulses in the upper-right corner, marked with legend following reference [Bibr bib1.bibx13]. Corresponding robustness with respect to the coupling J and the offset of the heteronucleus $\nu_{S}$ for the two rotation elements is simulated using the quality factor $\Phi_{\mathrm{PP}}$ , as defined in [Bibr bib1.bibx65]. $\Delta_{1}$ is set to 2.583 ms [Bibr bib1.bibx65].

In Fig. [Fig F1]A, the general scheme of all mentioned bilinear rotation elements is shown. While flanking pulses make up the difference in the various bilinear rotations, the central refocused delay of overall duration 1/J is common to all of them. It is in all cases this central element that is responsible for the distinction of the I and IS spin systems. With central 180 ${}_{x}^{\circ }$ pulses on both nuclei, the refocused delays provide a $\pi_{x}$ rotation for the uncoupled I spin and a $\pi_{y}$ rotation if the IS spin system is coupled with the matched heteronuclear coupling J . It is thereby important to note that the rotation transforms all three Cartesian components in a defined way.

Understanding this common design principle of all bilinear rotations, it is sufficient to make the central refocused delay robust in the desired way to significantly enhance all different elements at the same time. As a result, the central blocks derived in the COB-BIRD [Bibr bib1.bibx65] can directly be used to make any type of basic bilinear rotation robust in the J -coupling range of 120–250 Hz and an offset range determined by the used shaped pulses, i.e., 37.5 kHz for the previously reported J -compensated BUBI [Bibr bib1.bibx12] and BUBU [Bibr bib1.bibx14] pulse sandwiches, respectively. As the flanking pulses of a conventional BIRD element are 90° pulses, corresponding pulses need to be eliminated from the COB-BIRD element. We then obtain a robust universal-rotation-type element that equally rotates all remote protons by 180° around x and all directly bound protons by 180° around y . The resulting COB central element can be used for the construction of the BIRD, TANGO, BANGO, and BIG-BIRD elements (Fig. [Fig F1]B, D, and F), as shown in Fig. [Fig F1]C, E, and G. Together with the sequences, the corresponding offset vs. coupling profile of the general central element of the conventional bilinear rotations (Fig. [Fig F1]H), as well as the central COB element (Fig. [Fig F1]I), are given.

## 3 Construction principle of COB-enhanced bilinear rotations

Having derived the central necessary condition to make bilinear rotations more robust, we can also use it to construct highly compensated central π -rotation elements from already existing robust INEPT-type transfer elements. Considering just the initial pulse and the coupling evolution of such elements, they can be considered to be a J -selective transfer element leading to $I_{z}\to I_{x}$ for a spin without heteronuclear coupling and to $I_{z}\to 2I_{y}S_{z}$ for a matching J=1/(2Δ) -coupling constant. This behavior can be considered a point-to-point transformation, with an effective flip angle of $(\pi /2{)}_{y}$ for a spin without coupling and an effective flip angle of $(\pi /2{)}_{-x}$ for the coupled case in the coupling Hamiltonian $H_{J}=2\pi JI_{z}S_{z}$ . As long as only π pulses are applied and no mixing of spin states is apparent on the S spin, this transformation can be considered homomorphous or even isomorphous to a point-to-point pulse, transforming $I_{z}\to I_{y}$ with respect to its rotational structure (see Fig. [Fig F2]A).

**Figure 2 F2:**
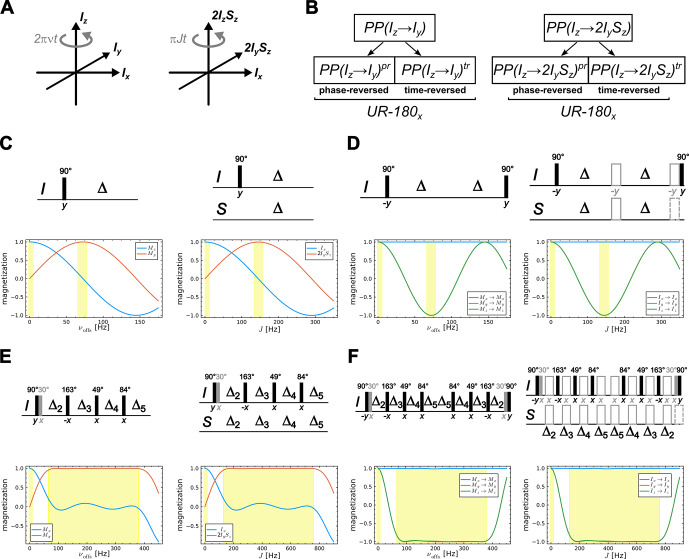
Construction principle for obtaining a bilinear rotation element from an INEPT-type transfer element. The principle uses the equivalent of a heteronuclear coupled two-spin system **(A)** to an offset selective shaped pulse **(A')** as both interactions describe a rotation in a 3D Hilbert (sub)space. The previously reported construction principle from an effective point-to-point π/2 pulse to a universal π rotation [Bibr bib1.bibx41] **(B)** can then be extended to construct a BIRD bilinear rotation from a reduced INEPT sequence following essentially the same construction procedure **(B')**. A simple selective excitation scheme (**C**, top), equivalent to an on-resonant, reduced INEPT element (**C'**, top), will then be constructed to give a selective π rotation (**D**, top) and the corresponding bilinear rotation (**D'**, top), respectively. Introducing a pair of 180° pulses (**D'**, drawn in gray) results in the offset-compensated classical scheme. Corresponding offset and J dependencies of excited coherences and magnetization transfers are given below the sequences. Note that the factor 2 in the offset vs. J dependence results from the rotation frequency of the two interactions. Using a more elaborate INEPT-type transfer element like the reduced COB3-INEPT [Bibr bib1.bibx13] **(E, E')** highly J -compensated bilinear rotations can be constructed **(F, F')**. Note that 30° pulses (drawn in gray) are added to the original COB3-INEPT sequence to ensure the transfer $I_{z}\to I_{x}$ for vanishing offsets or J couplings, respectively **(E, E', F, F')**. Delays are taken from [Bibr bib1.bibx13] to be $\Delta_{2}=4.221$ , $\Delta_{3}=2.0634$ , $\Delta_{4}=2.14$ , and $\Delta_{5}=1.075$ ms.

The target for the BIRD bilinear rotation, on the other hand, are overall π rotations represented by the propagator $U_{T}=\mathrm{exp}(-i\pi 2I_{\gamma_{I}}S_{\gamma_{S}}$ ), with $\left\{\gamma_{I},\gamma_{S}\}=\{x,x\right\}$ for two uncoupled spins I and S , and {y,y} for the coupled IS spin system. Again, this can be translated to a homomorphous frequency-selective universal rotation pulse with target propagators $U_{T}(0)=\mathrm{exp}(-i\pi I_{x})$ and $U_{T}(J)=\mathrm{exp}(-i\pi I_{y})$ for the rotational structure, as long as we are only interested in the effective I spin rotations. As it turns out, a construction principle exists, for which such π pulses can be created from corresponding π/2 pulses: for an effective universal rotation pulse with flip angle 2β , a time- and phase-reversed and an original point-to-point pulse with effective flip angle β starting from $I_{y}$ need to be applied consecutively to obtain the desired rotation [Bibr bib1.bibx41]. In the case of a point-to-point pulse transferring $I_{z}$ to $I_{y}$ at a defined offset ν , this can be reformulated to first apply the phase-reversed point-to-point pulse, followed by the time-reversed point-to-point pulse to construct a universal rotation $\pi_{x}$ pulse at offset ν (Fig. [Fig F2]B, left). Using the homomorphous/isomorphous relation to the INEPT-type transfer element and heteronuclear coupling evolution, a corresponding bilinear π rotation element can be constructed (Fig. [Fig F2]B, right).

Using the homomorph of the first pulse and the delay of a conventional INEPT element, which leads to π/2 rotations with the offset-dependent phase (Fig. [Fig F2]C, left), this can be used to construct a frequency-selective universal π rotation element (Fig. [Fig F2]D, left). This element directly translates into the coupling evolution case, for which the original INEPT-derived transfer element directly creates a conventional BIRD element. All it needs is the insertion of two pairs of 180° pulses to give the BIRD element the needed robustness for the chemical shift offsets (Fig. [Fig F2]D, right). In Fig. [Fig F2]E and F, the very same exercise is accomplished using the highly J -compensated COB3-INEPT sequence previously published in [Bibr bib1.bibx13]. The original COB3-INEPT was optimized only for the transfer $I_{z}\to 2I_{y}S_{z}$ for a given heteronuclear coupling constant J and the condition 120Hz≤J≤750Hz . The transfer $I_{z}\to I_{x}$ for the case J=0 Hz, however, was not taken into account. This is easily corrected by an additional 30 ${}_{x}^{\circ }$ pulse right after excitation, which compensates the sum of all other pulses applied on-resonant and without coupling being present. The overall COB3-BIRD bilinear rotation sequence looks complex, but a very high J compensation is achieved. Because many pulses are applied during the sequence, it should be noted that only the use of well-compensated shaped pulses (provided in [Bibr bib1.bibx13]) will lead to the desired result in experiments for a given range of chemical shift offsets.

We also applied the construction principle to the original COB-INEPT [Bibr bib1.bibx11] and the second broadband J -compensated COB3-INEPT sequence given in [Bibr bib1.bibx13], resulting in the sequences that are referred to as COB-BIRDcp and COB3-BIRDcp in the experimental verification section.

## 4 Performance with respect to J

To verify the validity of the various COB-enhanced bilinear rotation elements of Fig. [Fig F1], we recorded J -dependency profiles for four example bilinear rotations (BIRD, TANGO, BANGO, and BIG-BIRD) for different central inversion elements (Fig. [Fig F3]).

**Figure 3 F3:**
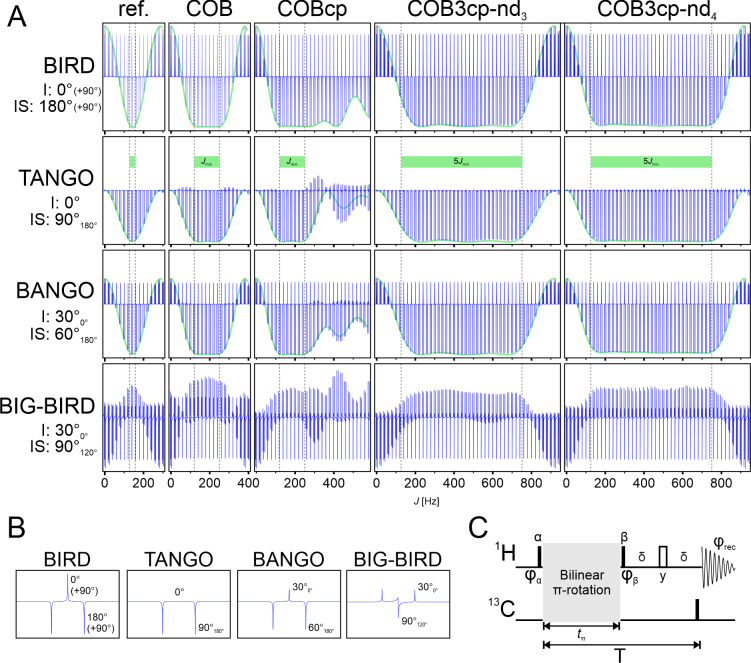
J dependencies of various example bilinear rotations. Experimental verification of the coupling profiles of the various bilinear π -rotation elements by their application in a BIRD, TANGO, BANGO, and BIG-BIRD pulse element. **(A)** Simulated coupling profile (green line) superimposed over the experimentally obtained data (blue spectral lines). Experimental data have been obtained from a 120 mM sample of sodium acetate (including 66 % sodium ^13^C_2_-acetate) in 4:1 DMSO- $d_{6}$ : D_2_O. From left to right, the spectra show the original sequences with refocused delays (ref. – delays matched to 145 Hz J coupling), the COB-BIRD (COB), the COB-BIRDcp (COBcp), the COB3-BIRDcp-nd3 (COB3cp-nd3), and the COB3-BIRDcp-nd4 (COB3cp-nd4) as the central spin system selective π rotations. From top to bottom, the respective elements are applied as a BIRD, TANGO, BANGO, and BIG-BIRD, with their respective flip angles for the *I* and *IS* spin systems shown. As the signal intensity of the BIG-BIRD is modulated by the phase, no simulation is superimposed. Within the TANGO series – using a green bar and dashed black lines – optimized regions of COB sequences are indicated. For display purposes, the last 90° pulse of the BIRD element has been left out as this would result in ±z magnetization. As the pulses are equal irrespective of the coupling, leaving it out does not affect the results – as can be seen from the correspondence between simulation and experimental data. **(B)** As an example of each pulse element, the matched case of the ref. spectrum is shown. The central line and outer signals correspond to the ^12^C_2_-acetate ( I ) and ^13^C_2_-acetate ( IS ), respectively. Respective flip angles for the pulse elements are indicated. **(C)** Relaxation-compensated pulse sequence used to record the J profiles. Pulses α , β with phases $\varphi_{\alpha }$ , and $\varphi_{\beta }$ are adapted to the different types of bilinear rotations, resulting in ( $90_{x}^{\circ }$ , $90_{-x}^{\circ }$ ) for the BIRD^*d*^, ( $45_{x}^{\circ }$ , $45_{x}^{\circ }$ ) for the TANGO, ( $60_{x}^{\circ }$ , $30_{x}^{\circ }$ ) for the BANGO, and ( $52.24_{129.23^{\circ }}^{\circ }$ , $135_{270^{\circ }}^{\circ }$ ) for the BIG-BIRD element. Variation in J is emulated by scaling the delays in the π rotation elements, as described in [Bibr bib1.bibx11], resulting in scaled overall π -rotation times $t_{\pi }$ . Relaxation compensation is achieved by adjusting the delay δ in such a way that the overall duration T stays constant.

While we varied the heteronuclear J coupling for simulations, the experimental incrementation of a coupling constant is not feasible, and we used varying delays instead to effectively measure the coupling dependence: as the evolution during all delays $\Delta_{i}$ is given by $\mathrm{cos}\pi J\Delta_{i}$ , an increasing coupling constant J can be also emulated by scaling all delays $\Delta_{i}$ with a common factor. In addition, we compensated potential signal losses due to increasing delays by a compensation delay after the bilinear rotation element, ensuring approximately identical transverse relaxation periods for all effective J couplings (Fig. [Fig F3]C). For the acquisition of J profiles, we used a 1:2 mixture of unlabeled acetate and ^13^C_2_-acetate with a measured coupling constant of ${}^{1}J_{\mathrm{CH}}=$ 125 Hz. The ratio of unlabeled vs. labeled acetate results in three lines of approximately equal intensities in an uncoupled ^1^H spectrum.

The resulting experimental J profiles are given in Fig. [Fig F3]A, with each row representing a different bilinear rotation element and each column representing a central π rotation element. We chose a BIRD^*d*,*X*^ (resulting in an initial 90 ${}_{x}^{\circ }$ pulse, while the second pulse is canceled, with an excitation pulse for the otherwise undetectable polarization), a TANGO (90^*I* *S*^, 0^*I*^) (with two 45 ${}_{x}^{\circ }$ flanking pulses), a BANGO with $\beta^{I}=$ 30° and $\beta^{IS}=$ 60° (using a 60 ${}_{x}^{\circ }$ pulse before and a 30 ${}_{x}^{\circ }$ pulse after the central element), and a BIG-BIRD sequence with $\beta^{I}=$ 30°, $\varphi^{I}=\;$ 0°, $\beta^{IS}=\;$ 90°, and $\varphi^{IS}=\;$ 120° (resulting in $\chi_{\varphi_{\chi }}=$ 52.24°_129.23^∘^_ and $\mu_{\varphi_{\mu }}=$ 135°_270^∘^_). In all cases but the BIG-BIRD profiles, we overlaid simulated J profiles as solid green lines over the experimental data for inner (uncoupled) and outer (coupled) multiplet components.

The central π -rotation elements used for the different J profiles are summarized in Fig. [Fig F4]D–H. The original spin system selective π rotation is based on a refocused delay of duration 1/J . The π rotation of the recently reported COB-BIRD [Bibr bib1.bibx65], as derived in Fig. [Fig F1], constitutes the second element. Further π rotations use the construction principle with previously derived COB-INEPT [Bibr bib1.bibx11] and COB3-INEPT [Bibr bib1.bibx13] building blocks, as described in the previous section and in Fig. [Fig F2]. The resulting elements are called COBcp, COB3cp-nd_3_, and COB3cp-nd_4_, where cp indicates the use of the construction principle and nd_*i*_ is the number of delays of the original INEPT-type building block.

**Figure 4 F4:**
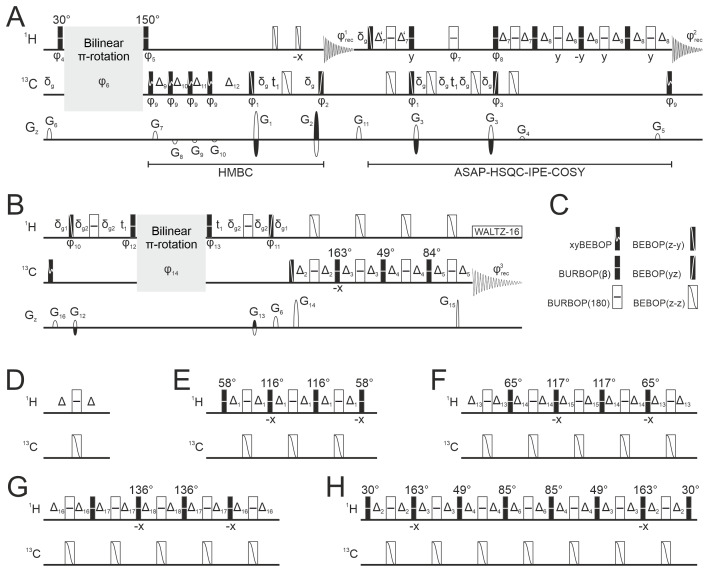
Detailed representation of the pulse sequences used in this article. **(A, B)** The full pulse sequences of the NORD supersequence **(A)** and the {^1^H} $,{}^{13}$ C- J -INEPT **(B)** where the gray box is replaced by the respective bilinear π -rotation elements **(D–H)**. The individual bilinear rotations are **(D)** BIRD, **(E)** COB-BIRD, **(F)** COB-BIRDcp, **(G)** COB3-BIRDcp-nd_3_, and **(H)** COB3-BIRDcp-nd_4_ (nomenclature explained in the main text). All pulses are applied as shaped pulses with their respective identification presented in **(C)**. If no flip angle is annotated above the BURBOP( β )-type pulses, a 90° flip angle is meant. All phases are along x, unless indicated otherwise. Phases are cycled following $\phi_{1}=$ x , - x , $\phi_{2}=$ x , - x , - x , x , $\phi_{3}=$ x , x , - x , - x , - x , - x , x , x , $\phi_{4}=$ x , $\phi_{5}=$ 4( x ), 4(- x ), $\phi_{6}=$ 4( y ), 4( x ), $\phi_{7}=$ x , y , x , y , y , x , y , x , $\phi_{8}=$ x , - x , x , - x , - x , x , - x , x $\phi_{9}=$ x , y , - x , - y , x , - y , - x , y , $\phi_{10}=$ 4( x ), 4(- x ), $\phi_{11}=$ 8( x ), 8(- x ), $\phi_{12}=$ y , - y , $\phi_{13}=$ - y , y , $\phi_{14}=$ x , x , - x , - x , $\phi_{\mathrm{rec}}^{1}=$ x , x , - x , - x , - x , - x , x , x , $\phi_{\mathrm{rec}}^{2}=$ x , x , - x , - x , and $\phi_{\mathrm{rec}}^{3}=$ 4( x ), 8(- x ), 4( x ), where the phase programs of $\phi_{6}$ and $\phi_{14}$ are applied on all proton pulses of the bilinear π rotation on top of the relative phases within the element. The delays correspond to $\Delta_{1}=$ 2.583 ms, $\Delta_{2}=$ 2.1105 ms, $\Delta_{3}=$ 1.0317 ms, $\Delta_{4}=$ 1.07 ms, $\Delta_{5}=$ 0.5375 ms, $\Delta_{6}=$ 2 ⋅ $\Delta_{5}=$ 1.075 ms, $\Delta_{7}=\;1/(4\cdot^{1}J{}_{\mathrm{CH}})$ , $\Delta_{8}=$ $>1/(4\cdot J_{\mathrm{HH}})\approx$ 30 ms recommended, $\Delta_{9}$ , $\Delta_{10}$ , and $\Delta_{11}$ are the three-fold low pass J -filter delays, $\Delta_{12}\approx 1/(2\cdot^{3}J_{\mathrm{CH}})$ , which was matched to an 8 Hz coupling, $\Delta_{13}=$ 0.394 ms, $\Delta_{14}=$ 2.134 ms, $\Delta_{15}=$ 2.983 ms, $\Delta_{16}=$ 0.5401 ms, $\Delta_{17}=$ 1.065 ms, and $\Delta_{18}=$ 2.1404 ms. In the ASAP-HSQC, the generalized Ernst-angle excitation is achieved by scaling $\Delta {’}_{7}=(\beta /90^{\circ })\Delta_{7}$ for an effective β -flip angle [Bibr bib1.bibx50]. The gradients used in the sequences are applied at the following strength relative to the maximum available gradient of approximately 50 G cm^−1^: $G_{1}=$ (79 %, - 47.3 %), $G_{2}=$ ( - 47.3 %, 79 %), $G_{3}=$ (41.5 %, - 41.5 %), $G_{4}=$ 5.2 %, $G_{5}=$ 15.7 %, $G_{6}=$ 31 %, $G_{7}=$ 23 %, $G_{8}=$ - 11 %, $G_{9}=$ - 7 %, $G_{10}=$ - 5 %, $G_{11}=$ 37 %, $G_{12}=$ (20.9 %, - 20.9 %), $G_{13}=$ $-G_{12}$ , $G_{14}=$ 83 %, $G_{15}=$ $G_{14}$ , and $G_{16}=$ 19 %. All gradients are applied for 1 ms with a subsequent $\delta_{g}=$ 200 µ s recovery delay, except for the gradients $G_{12}$ , $G_{13}$ , and $G_{15}$ , which are applied at 750, 750, and 500 µ s, respectively. The $\{{}^{1}H\}{}^{13}C$ - J -INEPT sequence has been applied with ${}^{13}C$ detection while decoupling protons using the WALTZ-16 decoupling sequence.

Impressively, the resulting J profiles show the improved robustness with J for all COB-type elements. While the COB π rotation has excellent performance over the full coupling range of 120–250 Hz, all other sequences have similar performance over their entire coupling range, with few negligible dents in performance. The COB-BIRDcp performs well even over a range significantly exceeding the optimized coupling range, which is not the case for the other bilinear rotations constructed from the COBcp element. The COB3cp building blocks, on the other hand, perform incredibly well over the entire optimized coupling range of 120–750 Hz. Just for COB3cp-nd_3_, it should be noticed that performance in the range of 120–190 Hz is somewhat compromised.

## 5 Application in NORD-type supersequences

An area in which bilinear rotation elements become increasingly important are so-called fast-pulsing supersequences. Here, bilinear rotation elements other than BIRD play an important role in spin system selective excitation and polarization storage. The development started with the NORD (NO Relaxation Delay) sequence [Bibr bib1.bibx44] and has since then found several extensions [Bibr bib1.bibx59] based on a generalized Ernst-angle scheme [Bibr bib1.bibx33]. A very useful NORD supersequence involves an HMBC with a subsequent H2OBC [Bibr bib1.bibx44], which results in two separate spectra where one shows typical long-range HMBC correlations, while the second spectrum gives almost exclusively one-bond and two-bond ^1^H,^13^C correlations. For a fast acquisition of the two spectra, it is mandatory that only protons with long-range ^1^H,^13^C couplings are excited for the HMBC to retain the polarization of directly ^13^C-bound protons for the subsequent H2OBC. This exactly matches the TANGO/BANGO profile or, if specific phase settings are also required, the BIG-BIRD element. We implemented the original pulse sequence from [Bibr bib1.bibx44] but experienced several problems resulting in non-absorptive lineshapes in the H2OBC subspectrum. We therefore modified the sequence to not use the H2OBC but an ASAP-HSQC-IPE-COSY with the very same information content instead. This novel sequence is essentially an HSQC-COSY, which uses a CLIP-COSY [Bibr bib1.bibx35] for coherence transfer as in [Bibr bib1.bibx22] but also retains unused polarization during ^1^H,^1^H-coherence transfer periods. As a consequence, the perfect echo element [Bibr bib1.bibx56] is extended to an isotropic perfect echo [Bibr bib1.bibx23], which will be described in more detail in a separate publication. The ASAP-HSQC, on the other hand, is an established fast-pulsing sequence using the generalized Ernst angle and maintaining unused polarization [Bibr bib1.bibx50]. The sequence with all experimental details is given in Fig. [Fig F4]A.

In the conventional NORD approach, a BIG-BIRD bilinear rotation is used for spin system selective excitation, which, of course, would be good to be replaced by a more robust element. The BIG-BIRD sequence in the NORD-HMBC-H2OBC experiment is needed to compensate phase twists in the H2OBC subspectrum. The ASAP-HSQC-IPE-COSY, instead, does not need phase adjustment, and a BANGO element can be used. For obtaining spectra shown in Fig. [Fig F5], we used 60° BANGO and COB-BANGO elements for comparison on a test sample containing several compounds with triple bonds. It must first be stated that the HMBC-ASAP-HSQC-IPE-COSY supersequence leads to HMBC- and HSQC-COSY-type subspectra of a very high quality.

**Figure 5 F5:**
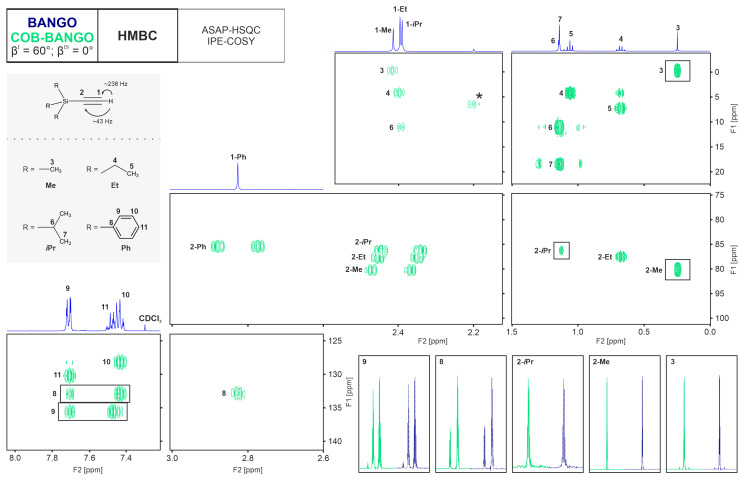
Experimental comparison of the HMBC subspectra extracted from the NORD supersequence when applying a BANGO or a COB-BANGO element for spin system selective excitation, as described in the main text. In the top-left corner, a schematic of the NORD sequence is shown, where the full pulse sequence is presented in Fig. [Fig F4]. The BANGO and COB-BANGO are designed to apply a 60° excitation for isolated I spins and store polarization along z for protons of IS spin systems for the subsequent ASAP-HSQC-IPE-COSY. The isotropic test sample consists of four silycic acetylenes, as shown in the gray box on the left, dissolved in CDCl_3_. The relevant regions of the HMBC applied with a COB-BANGO are zoomed in the six boxes and are aligned with each other and with corresponding 1D-^1^H regions on top. Corresponding carbon and proton assignments are given in the 2D regions and in the 1D-^1^H spectrum, respectively. Slices of selected boxed signals are given as representative examples on the bottom left with their respective ^13^C assignments. The solid green spectra and the dashed blue spectra correspond to HMBC slices of the COB-BANGO and BANGO versions, respectively.

Irrespective of the spin system selective excitation element, the Ernst-angle-type excitation as well as the application of the ASAP-HSQC sequence lead to very fast pulsing times without compromise in spectral quality and even improved sensitivity. In the direct comparison of the BANGO and COB-BANGO experiments, the HMBC (Fig. [Fig F5]) and the ASAP-HSQC-IPE-COSY (Fig. [Fig F6]) need to be compared separately.

**Figure 6 F6:**
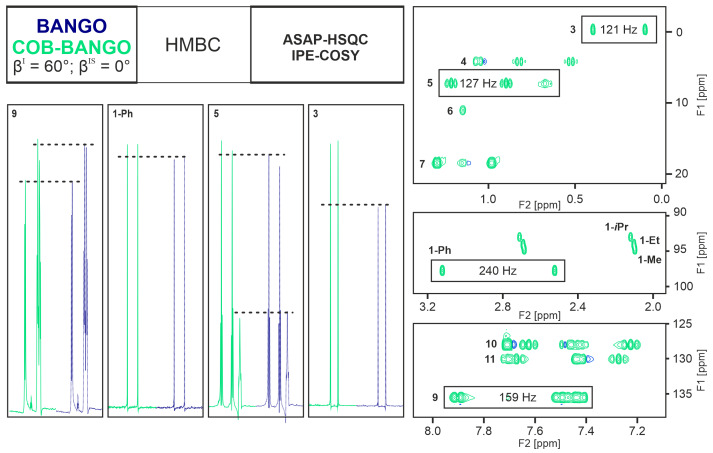
Experimental comparison of the ASAP-HSQC-IPE-COSY subspectra extracted from the NORD supersequence when applying a BANGO or COB-BANGO element for storing polarization throughout the HMBC sequence. At the top-left corner, a schematic of the NORD sequence is shown, where details of the sequence are given in Fig. [Fig F4]. The BANGO and COB-BANGO are designed to apply a 60° excitation for the I spins and storing polarization of the IS spins for the subsequent ASAP-HSQC-IPE-COSY. On the right side, 2D regions of the ASAP-HSQC-IPE-COSY are provided with the carbon assignments, as introduced in Fig.[Fig F5]. Slices of selected boxed signals are given on the left with their respective ^13^C assignment, whereas their respective ^1^J_CH_ couplings are annotated within the boxes.

The HMBC subspectrum in both cases is essentially identical. This behavior is expected, since the HMBC only uses polarization of I spins without direct coupling, and the excitation only relies on the performance of corresponding ^1^H pulses, which were all well-compensated optimal-control-derived shaped pulses. The situation is different for the ASAP-HSQC-IPE-COSY subspectrum, for which the bilinear rotation elements need fitting coupling constants. To already achieve best possible performance for the BANGO sequence, especially optimized universal rotation 30 and 150° shaped pulses, together with the BUBI refocusing and inversion pulse sandwich [Bibr bib1.bibx12], have been applied in combination with an overall delay matched to J=185 Hz. As a result, only slight improvements are expected for the COB-BANGO sequence for particularly mismatched coupling constants. Indeed, aromatic signals with approximately matching couplings do not show improvements worth mentioning. Furthermore, signals involved in triple bonds with a coupling of ≈ 240 Hz result in signal intensities improved only by about 5 %. Methyl groups, instead, show improvements exceeding 30 % signal intensity. We would have expected similar improvements for CH_2_ groups, but the signal with a multitude of homonuclear couplings and particularly the large ^2^J_HH_ coupling shows only a small enhancement on the order of 6 %. Still, the COB-BANGO in all cases shows better performance than the conventional BANGO, with well-compensated shaped pulses.

## 6 Application involving partially aligned samples

While conventional isotropic NMR samples very rarely show coupling constants outside the 120–250 Hz range, partially aligned samples with residual dipolar couplings on top of the scalar couplings can lead to total couplings easily exceeding the 120–250 Hz range. We therefore used the silycic acetylene compounds from the previous demonstration experiments and dissolved them in the lyotropic liquid crystal poly- γ -benzyl-L-glutamate (PBLG) with CDCl_3_ and measured ^1^H,^13^C one-bond couplings with our recently introduced COB- J -resolved INEPT experiment [Bibr bib1.bibx65]. The pulse sequence with experimental details is given in Fig. [Fig F4]B, where various BIRD-type elements derived from the π rotations of Fig. [Fig F4]D–H were used for homonuclear decoupling in the indirect dimension. The resulting cross peaks for the one- and two-bond heteronuclear correlations involving triple bonds are summarized in Fig. [Fig F7].

**Figure 7 F7:**
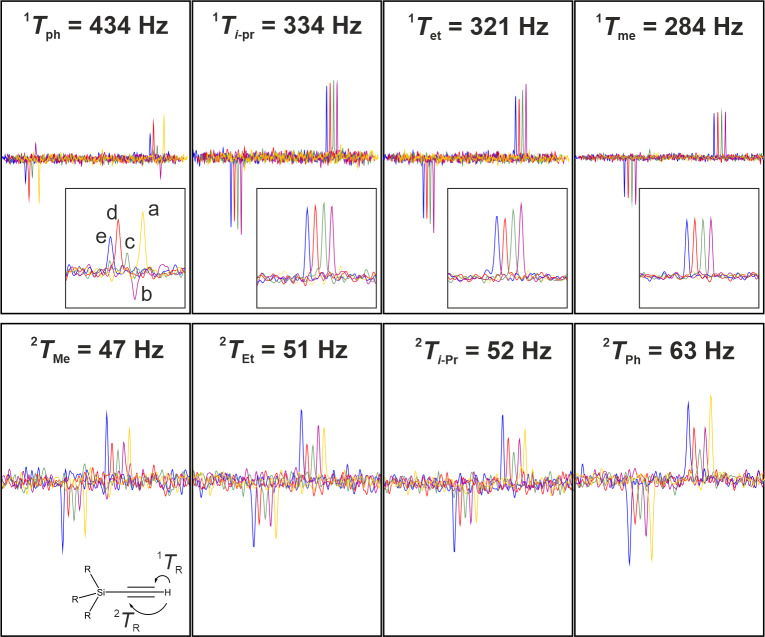
Comparison of acetylene signals for the various bilinear π -rotation elements using a J -resolved homonuclear-decoupled ^13^C-detected INEPT sequence. Shown are traces along the indirect J dimension with heteronuclear coupling evolution and ^1^H,^1^H homonuclear decoupling. Details of the pulse sequence are summarized in Fig. [Fig F4]B. For the measurement, the same silicic acetylene compounds as shown in Fig. [Fig F5] are dissolved in an 11 % lyotropic mesophase consisting of PBLG/CDCl_3_. Under partial alignment, total ${}^{1}T_{\mathrm{CH}}$ and ${}^{2}T_{\mathrm{CH}}$ couplings from scalar and residual dipolar contributions range from 47 to 434 Hz. The assignment and coupling size of each acetylene cross peak are indicated at the bottom left with the rest group (see gray box in Fig. [Fig F5]). For the comparison, the sequence was tested using **(a)** BIRD, **(b)** COB-BIRD, **(c)** COB-BIRDcp, **(d)** COB3-BIRDcp-nd_3_, and **(e)** COB3-BIRDcp-nd_4_ bilinear rotations for homodecoupling. The BIRD filter was matched to 145 Hz transfer delays – which happens to result in a perfect transfer for 145 Hz and 3⋅145 Hz = 435 Hz. For covering all couplings with the best possible intensities, the delays for the COB-BIRD, COB-BIRDcp, COB3-BIRDcp-nd_3_, and COB3-BIRDcp-nd_4_ are scaled relative to the values given in the caption of Fig. [Fig F4] by 0.75, 0.75, 1.25, and 1.25, respectively.

The conventional BIRD with delays set for 145 Hz works well for small couplings and, by accident, the largest coupling with ${}^{1}T_{\mathrm{CH}}=434$ Hz ( ≈3⋅145 Hz, three times the nominal coupling constant of the BIRD element). For couplings in the range of 284–334 Hz, no cross peak is observed. In contrast, all COB-type BIRD elements result in cross peaks for all carbons. It should be noticed that all COB-type elements were scaled as described in the figure caption to provide best intensities for the entire 47–434 Hz range. For the largest coupling as well as for all small couplings, differences can be seen, with the COB3-type elements performing best.

## 7 Discussion

A thorough analysis of various bilinear rotation elements resulted in two design principles that allow us to produce all kinds of compensated bilinear rotations from an existing compensated bilinear rotation or even an existing compensated INEPT-type transfer element. The first principle is based on the finding that a common central π -rotation element is the essence of all basic types of bilinear rotations. By selectively optimizing this central element, this allows us to equally improve all bilinear rotations. In NMR spectroscopy, such robust π -rotation elements can be taken from existing ones, like the one derived for the original COB-BIRD [Bibr bib1.bibx65] or, for example, the CAGE-BIRD sequence for the compensation of homonuclear antiphase evolution [Bibr bib1.bibx23]. The second construction principle is an extension of a previously derived one for universal rotation pulses with flip angle 2 β that are combined from two point-to-point pulses with effective flip angles β [Bibr bib1.bibx41]. As we could show, this construction principle can be translated from offset-dependent single-spin pulse shapes to J -dependent transfer elements like INEPT as long as the elements are reduced to pulse on only one of the coupled spins. Instead of using such a construction element, of course, a central π -rotation element can also be optimized from scratch, for which we refer the interested reader to [Bibr bib1.bibx65].

All sequences introduced here have been applied with previously published shaped pulses, which have all been optimized for ^1^H,^13^C applications on a 600 MHz spectrometer, corresponding to a high-end system for typical small-molecule applications. If offset ranges different from 10 kHz on ^1^H and 37.5 kHz on ^13^C are desired, just shaped pulses may be re-optimized using standard optimal control procedures, for which several optimization programs are readily available [Bibr bib1.bibx9]. Be aware that the optimization of fully J -compensated pulse sandwiches like BUBI and BUBU [Bibr bib1.bibx12] may be quite demanding. If simple universal rotation pulses are instead applied simultaneously to the two heterochannels, a certain compromise in unspecified J -coupling evolution during the pulses must be expected. Pulse shapes may also be re-optimized if higher compensation for B_1_ inhomogeneity is needed.

If the sequences are to be transferred to other spins – for example, to ^1^H,^15^N – the J compensation can be scaled down to smaller J couplings by correspondingly extending the delays in the central part of the bilinear rotations. The COB-BIRD, for example, will work for a coupling range of 60–125 Hz (instead of 120–250 Hz) if all delays are doubled.

In actual applications, the COB-type bilinear rotations work very well for a wide variety of heteronuclear J and dipolar couplings. However, homonuclear couplings are not taken into account in the optimization process. Small ones are easily tolerated, but if large and many ^1^H,^1^H couplings are present, significant magnetization might be lost during the elements. Bilinear rotations generally only work well as long as the condition ${}^{1}J_{\mathrm{CH}}\gg \sum_{i}J_{\mathrm{HH},i}$ is sufficiently fulfilled. This is particularly an issue with CH_2_ groups with their large ${}^{2}J_{\mathrm{HH}}$ couplings and the potentially high number of additional couplings. Since COB-type sequences are significantly longer than the conventional doubly refocused delay, the loss of magnetization due to homonuclear couplings is also expected to be higher and easily explains the reduced gain observed for the CH_2_ group compared to the methyl group in the ASAP-HSQC-IPE-COSY of Fig. [Fig F6]. Equally, fast-relaxing molecules may experience a greater loss in sensitivity for the longer sequences. For a fair judgment of the different sequences, the duration should not be used directly to estimate the coupling evolution and relaxation effects. Due to odd pulse flip angles, part of the magnetization is also stored along z during the COB-type elements, reducing the strengths of the unwanted effects.

Regarding fully isotope-labeled samples, robust bilinear rotations based on the BASEREX approach [Bibr bib1.bibx24] may be obtained for a specific bandwidth if all carbon broadband pulses are replaced by corresponding shaped pulses with controlled coupling evolution like the previously used REBURP pulse shape [Bibr bib1.bibx24].

As compensated COB-type BIG-BIRD sequences are generally possible, corresponding compensated TIG-BIRD sequences [Bibr bib1.bibx7] can also be constructed. Spin state selectivity must be obtained with an additional transfer element, which will still have the restrictions of the conventional refocused delay approach. Only for special cases – for example, if pure antiphase magnetization for the IS spin system is targeted – equally compensated sequences like the COB-INEPT [Bibr bib1.bibx11] are available for the extension.

## 8 Conclusions

Highly compensated bilinear rotations of the BIRD, TANGO, BANGO, and BIG-BIRD type have been introduced with J compensation up to a J -coupling range of 120–750 Hz and offset ranges for ^1^H and ^13^C of 10 and 37.5 kHz, respectively. This has been achieved using a general principle, in which the central refocused delay common to all basic bilinear rotations is being optimized or constructed from existing elements. In particular, the latter has been achieved using a construction principle originally derived for shaped pulse design for single spins. Effective ways of producing bilinear rotations with other demands on J -coupling ranges or offset/B_1_ compensations are discussed.

We foresee direct applications for the different bilinear rotations introduced here in the area of partially aligned samples, where a large range of (J+D) couplings is unavoidable, or in NMR-service applications that also consider sp-hybridized carbons with typical ${}^{1}J_{\mathrm{CH}}$ coupling constants around 250 Hz. Another obvious field will be ^19^F-based correlation experiments, for which one-bond couplings show a relatively wide distribution, and many more potential applications are thinkable.

## Data Availability

Spectra in JCAMP-DX and Bruker format, together with Bruker pulse programs used for the acquisition of example NMR spectra, are available at 10.35097/kam044sfpb43ektf [Bibr bib1.bibx64] at KITOpen with identifier 1000187362.
